# Genomic insights into fragmentation and translocation in European green toads

**DOI:** 10.1016/j.isci.2026.115395

**Published:** 2026-03-17

**Authors:** Leonie Muriel Walderich, Alvin Wiwiet Susanto, Mikael Svensson, Anna Fohrman, Mats Wirén, Rachael O’Dwyer, Kristofer Försäter, Patrik Rödin-Mörch, Jacob Höglund

**Affiliations:** 1 Department of Ecology and Genetics, Uppsala University, Norbyvägen 18D, 752 36 Uppsala, Sweden; 2 Department of Biology, Lund University, Sölvegatan 35, 223 62 Lund, Sweden; 3 SLU – Swedish Species Information Centre, Box 7007, 750 07 Uppsala, Sweden; 4 County Administration Board of Skåne, Södergatan 5, 205 15 Malmö, Sweden; 5 Amplexus Naturkonsult, Grevegatan 20, 216 17 Limhamn, Sweden; 6 Foundation Nordens Ark, Åby Säteri, 456 93 Hunnebostrand, Sweden

**Keywords:** nature conservation, ecology, zoology, genetics

## Abstract

The European green toad (*Bufotes viridis*) is Sweden’s most threatened amphibian. Its range has contracted over the past century, with many local extinctions; remaining populations are fragmented and often isolated. Since the 1990s, conservation has focused on translocations to existing breeding sites and new localities, but many efforts have had limited success. We detected lower genetic diversity in Scandinavian populations (southern Sweden and nearby Denmark) than in Poland, plus strong structure and differentiation among Scandinavian subpopulations, implying unexpectedly low gene flow despite translocations. Small, isolated populations are strongly affected by drift, and whole-genome analyses reveal inbreeding and high genetic load in some subpopulations. We recommend reassessing source populations for translocations: the stock used in captive breeding and most past releases shows intermediate diversity but also signs of divergent selection and putative local adaptation. Management should balance minimizing inbreeding depression against risks of outbreeding depression and erosion of local adaptation risks.

## Highlights

• Swedish green toad populations are small, fragmented and endangered
• Translocations and reestablishment have had limited success
• The populations are genetically differentiated, inbred and have low diversity
• The source population for translocations is locally adapted and should be replaced

## Graphical abstract

**Figure:**
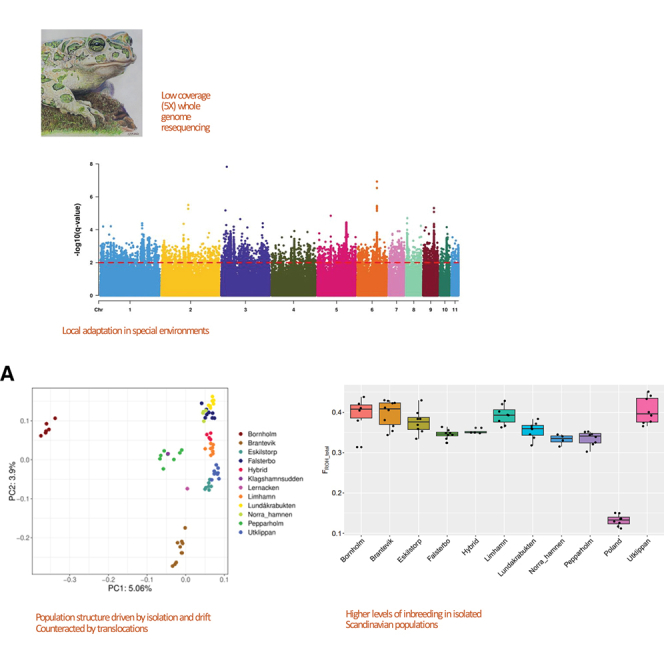

## Introduction

Demographic rescue of populations is often crucial for the persistence of local populations.^1^ This has prompted conservationists to engage in translocation projects to rescue populations at risk of local extinction. While some of these projects have claimed to be successful^2^^,^^3^ others have failed. It is likely that failed attempts are under-represented in the literature. In cases where demographic rescue has been established, the long-term persistence of any local population remains dependent on genetic diversity. Small populations commonly display low genetic diversity and may thus have reduced evolutionary potential, which can have long-term effects.^4^^,^^5^^,^^6^ Thus, translocation projects often aim at increasing local genetic diversity, a strategy termed “genetic rescue.”^7^ To assess if and why a translocation event is successful, it is crucial to consider the genetic composition of a population in addition to its size. Factors such as local adaptation and inbreeding can have an impact on demographic dynamics even after the numbers seem to recover following a decline.^5^ Population genomic analysis of genome-wide variation can support choices of source populations for translocations and captive breeding and determine where gene flow naturally exists and where human-mediation might be beneficial to avoid inbreeding.^8^

Small populations typically experience high levels of genetic drift and inbreeding.^9^^,^^10^ As effective population size decreases, the relative impact of the forces shaping genetic diversity changes.^11^ For small, highly fragmented populations, genetic drift and inbreeding becomes more impactful than selection.^12^^,^^13^ Both processes can cause recessive deleterious alleles to drift to high frequencies^14^ and in some cases become fixed.^15^ Conversely, other alleles are randomly lost through genetic drift leading to the decrease of adaptive evolutionary potential with negative long-term effects on species viability.^4^^,^^6^^,^^16^ While the efficacy of natural selection is anticipated to diminish with decreasing population size, it is yet difficult to predict at what population size and level of diversity future local adaptation becomes increasingly more difficult.^17^

Genetic load describes the proportion of standing genetic variation which have varying degrees of negative fitness effects. The phenotypic expression of genetic load can depend on the environmental context and associated gene expression patterns.^18^^,^^19^ Genetic load has two main components. The realized load encompasses deleterious alleles in homozygous state, as they become expressed they can also be purged by purifying selection. The masked load contains heterozygous deleterious alleles and can be maintained at low frequencies in a population due to mutation-selection balance.^19^^,^^20^ Low frequency completely recessive alleles are not purged as they are not expressed in the heterozygous state. As a threatened species’ population size decreases, both inbreeding and genetic drift convert parts of the masked load into realized load.^19^ When identical chromosomal segments are inherited from each parent, the offspring genome contains long stretches of homozygous genotypes.^20^ Homozygosity is not inherently harmful; however, if it occurs at loci with deleterious recessive alleles that become expressed, or if it decreases heterozygosity at loci with heterozygote advantage, homozygosity can cause negative fitness effects known as inbreeding depression.^19^^,^^21^^,^^22^^,^^23^ A further complication comes from the fact that alleles at any given locus may be locally adapted, thus allelic combinations that may have a fitness advantage in a donor population may confer a disadvantage in a recipient population and vice versa.^24^

Historically, inbreeding has been estimated using pedigree data; however, detailed parentage records over many generations are rarely available for wild species of conservation concern.^3^^,^^15^ The advancement of genomic analysis allows the detection of long runs of homozygosity (ROH) resulting from inbreeding.^15^^,^^25^^,^^26^ Szpiech and coworkers^27^ showed that ROH serve as a reservoir of homozygous deleterious variants. Based on the length and prevalence of ROH across the genome, the inbreeding coefficient F_ROH_ can be estimated to predict the risk of inbreeding depression with increased precision compared to pedigree data.^15^^,^^28^^,^^29^^,^^30^ Additionally, the length of ROH can inform us about the inbreeding history of populations.^31^ Long ROH indicate recent inbreeding, whereas shorter ROH suggests inbreeding in the more distant past where recombination has since broken up long ROH into shorter homozygous segments. Investigating inbreeding via ROH from whole-genome sequencing data is thus a powerful way to estimate the risk of inbreeding depression and inform genetic rescue actions.

Genomic erosion decreases the long-term survival of a species even after the population size has recovered and immediate threats have been removed.^4^^,^^5^^,^^16^ Severe genomic erosion as a direct consequence of a small effective population size *N*_e_ can contribute to a feedback loop in which inbreeding and genetic drift cause a reduction in fitness such as the production of fewer offspring, which further decreases N_e_, and ultimately leads to more loss of genetic diversity via increased genetic drift and inbreeding. This self-reinforcing process has been coined “the extinction vortex”^13^^,^^32^ and has been observed in several natural systems, for example, in populations of endangered shorebirds,^33^ Iberian lynx,^34^ and in a butterfly metapopulation.^35^

It is difficult for populations to break out of the extinction vortex and recover from genomic erosion. However, genomic monitoring and human-mediated gene flow through translocations can provide a chance for genetic rescue. One aim of translocations should be to improve connectivity and thus ensure ongoing gene flow between populations. However, if individuals from a large, genetically diverse population are added to a small population, it may introduce genetic load especially if the source population has high levels of heterozygosity.^36^ The introduced individuals might carry a large masked load that becomes expressed in the offspring generations due to continued inbreeding^19^^,^^23^ and/or introduce alleles not fit in the new environment.^24^ Nevertheless, careful genomics-informed choice of donor populations and the establishment of sustained gene flow can improve the longevity of threatened species.^37^

In this study, we aimed to use population genomic analysis to inform conservation efforts for the remaining green toad (*Bufotes viridis*) populations in Sweden. The European green toad is one of the most threatened amphibian species in Sweden.^38^^,^^39^ It has a broad distribution ranging from central Europe to Kazakhstan in the east, to southern Greece in the south.^40^^,^^41^^,^^42^ At the northern fringe of the distribution, only a few, small, isolated populations remain in about 10 locations in southern Sweden.^39^ Past studies have shown that the Swedish populations harbor lower genetic variation in both putatively neutral and potentially adaptive immunogenetic markers compared to populations more central to their distribution.^43^ These results align with the biogeographic trend of a reduction in intraspecific genetic diversity with increasing latitudes^44^^,^^45^^,^^46^^,^^47^ as well as the central-marginal hypothesis, which states that genetic diversity decreases toward the periphery of a species distribution.^48^ Therefore, the northern green toad populations are predicted to be more vulnerable and less adaptable to environmental changes compared to their southern counterparts.

From 2000 to 2015, the green toad was listed as critically endangered in Sweden as land use change, road networks, altered ground water levels, competition by the common toad (*Bufo bufo*), and predation by grass snakes (*Natrix natrix)* were drastically reducing population numbers.^49^ An action plan for the conservation of green toad was implemented by the County Administrative Board of Skåne and since 1994 over 180,000 green toads of various life stages have been translocated, or released from a captive breeding population at Nordens Ark Zoo in Västra Götaland and in total more than 430,000 eggs, 630,000 larvae, 57,000 juveniles, and approximately 3,700 sub-adults and adults have been used to boost and re-establish populations.^39^ The conservation status was changed to vulnerable in 2015 as population numbers seemed to increase. Unfortunately, many translocations were not successful and populations decreased again between 2016 and 2020. The current estimated population size is 750 reproductive females and 1,500 males, which is only 50% of the action plan goal to ensure a stable meta-population.^38^^,^^39^^,^^50^

This study focused on the remaining, small subpopulations of green toads in southern Sweden and nearby Denmark. We used low-coverage whole-genome sequencing data to estimate the level of genetic diversity, inbreeding, and mutation load within the Swedish and nearby Danish subpopulations and compared them to a Polish subpopulation. As fringe populations at high latitudes, we expected the Swedish and Danish subpopulations to have lower genetic diversity compared to the Polish. We investigated population structure to better understand genetic differentiation between the subpopulations, determine current gene flow, and the potential for connectivity to inform habitat conservation and future reintroductions. Due to the limited dispersal radius of the green toad and the fragmented nature of the remaining subpopulations, we expected to find a high degree of differentiation, possibly including local adaptations. We analyzed inbreeding signatures in the form of genome-wide ROH to evaluate the choice of the captive breeding source population and assess the risk of inbreeding depression. We anticipated to find overall more and longer ROH from more recent inbreeding in Swedish and Danish subpopulations compared to the Polish, due to isolation and small population sizes. Finally, we looked for evidence of local adaptation and elevated population differentiation between the population used for the breeding program, as well as most translocations, and the other remaining Swedish populations to examine whether the chosen source population is the most adequate one to be used in future conservation efforts.

## Results

### Recorded translocations

Despite repeated efforts, both the demographic and genetic success of attempted translocations have been limited. Supposedly, 18 localities in the area covered in this study have been subjected to translocations.^39^ Two new localities have been established (Brantevik and Lernacken). However, only Brantevik is considered successful so far. There have also been translocations among established breeding populations (Table S1), one of which most likely saved the Utklippan population. In the late 1980s, no males could be found at that site, only a few females. In 1994, 10 adult males and 100 yearling toads from Limhamn or Eskilstorp (records are uncertain) were translocated to Utklippan. The first reproduction since the support release was discovered in 1997, and in 2003 a total of 356 adults were recorded. In 2017, it was estimated the population size was more than 1,500 adults. The population has since crashed and the latest census in 2025 recorded 130 females and 420 males (Mats Wirén pers. obs.).

### Population structure

**Figure 1:**
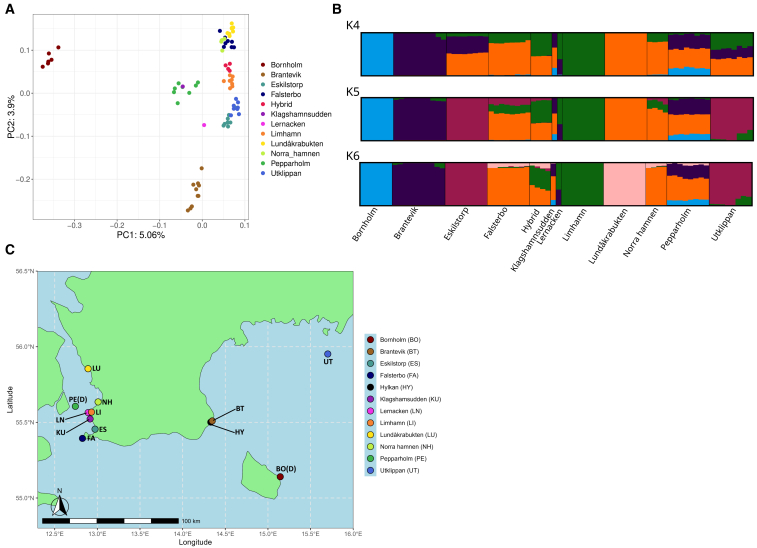
Population structure (A) PCA of samples from Sweden, and Bornholm and Pepparholm in Denmark plus the hybrid offspring from crosses of parents from Limhamn and Norra hamnen. (B) Admixture plot showing ancestry proportions of individual samples from the same populations at *K* = 4–6. (C) Scandinavian sampling locations of European green toad samples used in the study (sites in Denmark marked with D). The Swedish sites correspond to all known present locations of the species in Sweden. Details on each site is provided in Table 1.

In principal-component analysis (PCA), PC1 separated the Polish population from the other samples and captured 14.12% of the variation and along PC2, Bornholm was clearly separated from the rest of the samples (Figure S1A). After removal of the Polish samples, Bornholm was still separated and PC1 captured 5.06% of the variation (Figure 1A). After removal of both the Poland and Bornholm populations, PC1 accounted for 4.37% of the variation. The populations showed clear differentiation with the exception of Norra hamnen and Falsterbo clustering together and the one sample from Klagshamnsudden that clustered with Pepparholmen. (Figure S2A). The individuals experimentally created by crossing Norra hamnen and Limhamn parents (henceforth termed hybrid) clustered in between the parental populations as expected (Figure S2A).

**Table 1:** Information on the Scandinavian samples sequenced in this study

Location	Individuals	Sampling year	Area description
Utklippan	8	2020	ponds on a small island in the Baltic sea, supplemented
Lundåkrabukten	8	2020	shore meadow area close to the Saxån river, supplemented
Limhamn	8	2020	an abandoned limestone quarry near Malmö, source for breeding program and releases. Supplemented
Norra hamnen	4	2020	industrial area in Malmö, spontaneous
Eskilstorp	8	2021	grazed shore meadow, source for releases. supplemented
Falsterbo	8	2021	ponds in a golf course, supplemented
Brantevik	8	2024	coastal pond near a residential area, re-established by translocations
Hylkan	2	2024	ponds close to the Brantevik site, immigration from Brantevik
Lernacken	1	2024	ponds located close to the Øresund bridge, established by translocations
Klagshamnsudden	1	2024	coastal ponds, established by translocations
Pepparholmen	8	2024	ponds on an artificial island built as part of the Øresund bridge construction, spontaneous
Bornholm	6	2024	coastal ponds on the Danish island in the Baltic, natural

For locations, see map in Figure 1.

The NGSAdmix analysis for the entire dataset suggests the optimal *K* = 6 (Figure S1C). Poland and Bornholm were clearly differentiated when compared to the rest of the populations. After removing these two, there was still evidence of differentiation among the rest of the populations as the NGSAdmix analysis suggests *K* = 5 (Figure S1D) as the best number of clusters describing structure among the Scandinavian populations. When Poland was excluded, we observe five distinct populations (Bornholm, Brantevik, Eskilstorp with Utklippan, Limhamn, and Lundåkrabukten) whereas the rest of the locations appear admixed at varying degrees (Figure 1B). Thus, these analyses confirm the results obtained from PCAs.

The interpretation of *K* = 4 is similar to *K* = 5 with the exception of Eskilstorp/Utklippan now showing admixture. At increased resolution (*K* = 6), Lundåkrabukten appear distinct. The experimentally bred hybrids between Limhamn and Norra hamnen appear admixed for all values of *K*, as expected (Figure 1B).

The pairwise F_ST_ values range from 0.03 to 0.30 (Figure S3). All pairwise comparisons with the Polish and the Bornholm populations show values between 0.24 and 0.30. Among the Scandinavian populations, Lernacken, hybrids, Pepparholmen, Eskilstorp, Falsterbo, and Lundåkrabukten show relatively low genetic differentiation (F_ST_ < 0.1) while comparisons involving Limhamn yield high F_ST_ except when compared with Utklippan and Brantevik.

### Genetic variation and deviations from neutrality

Nucleotide diversity (θ_π_) and heterozygosity (H_o_) is highest in the Polish population while all other subpopulations show similarly lower levels with the lowest estimates observed in Bornholm and Brantevik. There is overall low variation among the Scandinavian subpopulations. The mean values of nucleotide diversity (θ_π_) range from 1.1 × 10^−3^ to 2.7 × 10^−3^ and heterozygosity range from 0.0007 to 0.0015 (Table 2). The estimates based on segregating sites—Watterson’s θ_W_, are very similar to nucleotide diversity θ_π_, and show similar patterns with large standard deviations among populations. Thus, Tajima’s “D” do not deviate significantly from “0,” indicating no dramatic deviations from mutation-drift expectations.

**Table 2:** Nucleotide diversity θ_π_, genetic diversity based on segregating sites—Watterson’s theta θ_W_, mean Tajima’s D (T_D_) statistics, average observed heterozygosity (H_o_), and their associated standard deviations across the chromosomes in the twelve green toad populations

Population	*n*	θ π	θ π SD	θ_W_	θ_W_ SD	T_D_	T_D_ SD	H_O_	H_O_SD
Bornholm	6	0.0011	0.0203	0.0010	0.0168	0.1105	0.8717	0.0007	0.0002
Brantevik	8	0.0014	0.0231	0.0011	0.0171	−0.023	0.9984	0.0007	0.0001
Eskilstorp	8	0.0013	0.0214	0.0012	0.0166	−0.0385	0.8392	0.0009	0.0001
Falsterbo	8	0.0014	0.0227	0.0013	0.0176	−0.0139	0.8645	0.0009	0.0001
Hybrids	4	0.0012	0.0210	0.0012	0.0182	0.0459	0.6316	0.0010	0.0002
Lernacken	1	0.0013	0.0257	0.0013	0.0247	0.2698	0.4007	0.0015	0.0002
Limhamn	8	0.0012	0.0212	0.0011	0.0164	−0.026	0.8806	0.0008	0.0001
Lundåkrabukten	8	0.0012	0.0208	0.0011	0.0163	0.0012	0.9103	0.0008	0.0001
Norra hamnen	4	0.0012	0.0208	0.0011	0.0180	0.0988	0.6509	0.0010	0.0002
Pepparholm	8	0.0015	0.0243	0.0013	0.0189	−0.0236	0.9278	0.0008	0.0001
Poland	8	0.0027	0.0306	0.0025	0.0247	−0.0858	0.7072	0.0019	0.0002
Utklippan	8	0.0012	0.0197	0.0012	0.0169	−0.2062	0.928	0.0012	0.0002

### Inbreeding

**Figure 2:**
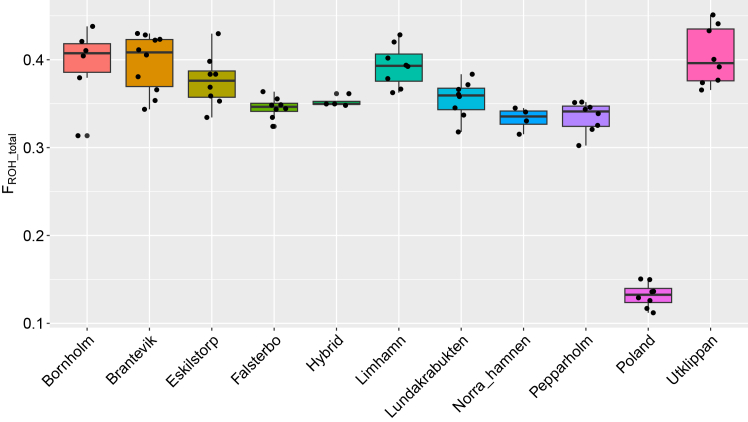
Boxplots of total F_ROH_ for each population (line through the box indicate the median, boxes describe 25–75 percentiles, outlier values are given as points)

Out of all populations, the Polish samples have the lowest mean inbreeding coefficient. While Bornholm, Brantevik, and Utklippan have the highest F_ROH_. Overall, the mean F_ROH_ values were similar across Scandinavian populations with the lowest value observed in Norra hamnen (Figure 2).

For ROH larger than 1 Mb, indicating recent inbreeding, the Polish population has the lowest inbreeding coefficient, F_ROH_ being close to zero, whereas Limhamn and Brantevik have the highest. Overall, the F_ROH_ values of the other populations are comparable. Among the Scandinavian populations, Norra hamnen shows the lowest F_ROH_ (Figure S4A).

For shorter ROH (<500 kb), indicating historical inbreeding, the Polish samples again have the lowest mean inbreeding coefficient, F_ROH_ being close to zero, while the island populations Utklippan and Bornholm have the highest (Figures S3B and S3C).

### Genetic load

**Figure 3:**
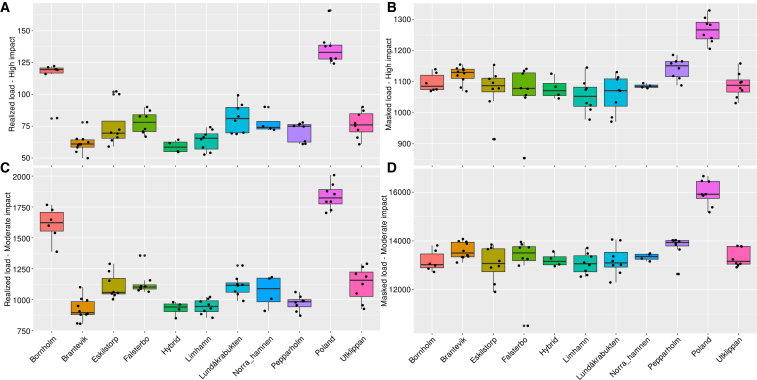
Boxplots showing genetic load (A–D) Realized and masked load in each population (line through the box indicate the median counts, boxes describe 25–75 percentiles, outlier values are given as points). (A) Counts of realized high impact variants, (B) masked high impact variants, (C) realized moderate impact variants, and (D) masked moderate impact variants.

As expected, the masked load (Figures 3A and 3C), regardless if highly deleterious or moderately so, is an order of magnitude higher than the realized load (Figures 3B and 3D). Furthermore, high impact mutations are less common than moderate impact mutations for both categories. The Polish population, which is larger and therefore harbors more variation than the Scandinavian, shows the highest masked and realized load for both impact categories (Figure 3). Among the Scandinavian populations, the isolated island population of Bornholm, which has the lowest diversity shows the second highest realized load for both impact categories, whereas Brantevik, Limhamn, hybrids, and Pepparholmen show the lowest realized load (Figures 3A and 3C). The high impact masked load is also highest in Poland and shows similar levels within Scandinavia, with the exception of Pepparholm and Brantevik, which show slightly elevated levels (Figure 3B).

In terms of the ratio of high impact realized load to synonymous mutations (low impact), we find that Poland has the lowest ratio for both realized and masked load and that there are no striking differences in realized load among Scandinavian populations (Figure S5A). For masked load however, we do find elevated levels in the two isolated island populations of Bornholm and Utklippan (Figure S5B). For both counts and ratios of moderate impact mutations to synonymous mutations, we find qualitatively similar patterns as for high impact mutations (Figures 3 and S5).

### Evidence of putative divergent selection in Limhamn

**Figure 4:**
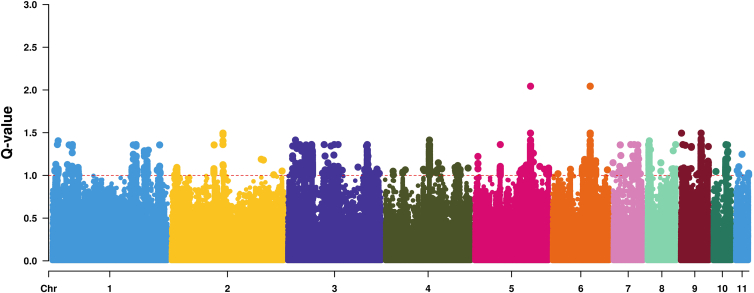
Manhattan plot of the outlier analyses of Limhamn compared to all other Swedish populations (i.e., Polish, Bornholm and hybrid samples excluded). The red line indicates a significance level of 0.01

When comparing Limhamn to the other Scandinavian populations (Bornholm, Poland and hybrids excluded), the selection scan identifies a total of 11,578 variants under putative divergent selection (*q* value <0.0L, Figure 4). These variants overlapped with 123 annotated protein coding genes and their gene ontology terms were significantly enriched for biological processes involved in wound healing, embryonal development (epiboly) and morphogenesis (Table S2).

## Discussion

### Genomic diversity

Overall, we observe lower genetic diversity in the Scandinavian populations compared to the Polish. This suggests that there might be low adaptive potential in the Scandinavian populations and confirms findings of a previous study based on ddRAD of Polish and Swedish samples.^51^ Höglund and colleagues^43^ also found lower genetic diversity of immune genes and neutral microsatellite markers in northern green toad populations. The central-peripheral and leading-edge effect hypotheses predict that smaller, fragmented populations would have smaller effective population size and increased geographical isolation, as well as harsher ecological conditions leading to lower genetic diversity.^48^^,^^52^ It is generally challenging to disentangle these factors and the green toad has likely been experiencing a combination of latitudinal and peripheral gradient effects throughout the recolonization process following the last glacial maximum 20,000 years ago. The observed natural populations are expected to have resulted from multiple founder events during the expansion to Sweden from the Middle East through central and Eastern Europe, which reduced genetic variation and increased impact of genetic drift on small, isolated northern populations.^43^ Additionally, increased anthropogenic land development and expansion of road networks has contributed to habitat fragmentation and subpopulation isolation more recently, further inhibiting gene flow.^49^

### Population differentiation and admixture

Since the start of the species action plan for Swedish green toad populations in the early 1990s, a total of 430,000 eggs, 630,000 tadpoles, and 3,700 adult toads have been released into the wild to boost existing populations and establish new ones within their historic range.^39^ The project is an ongoing, large conservation effort involving the Swedish Environmental Protection Agency, various county administrative boards and a large-scale captive breeding program at Nordens Ark Zoo. Genetic information alone is rarely sufficient to save a threatened species; underlying ecological problems exacerbating the decline must also be addressed. However, genomic data can be utilized to prioritize resource allocation and optimize conservation efforts. Additionally, genomic analyses can be used to design population models and simulate various demographic and ecological scenarios to extrapolate to other populations and estimate minimum viable population sizes for more accurate predictions about long-term population dynamics.^16^

Both the PCA and admixture analysis showed that there is clear differentiation between the Polish and Bornholm populations and the rest of the sampled Scandinavian populations. Furthermore, the clear genetic structure among many of the Scandinavian green toad subpopulations indicates a limited success of past releases. Despite the many thousands of potential recruits, if the support releases had led to successful reproduction, we would have expected to see higher levels of admixture and less population structure. The differentiation observed is likely a result of strong genetic drift in small and isolated populations with very low or no gene flow. The historical meta-population structure has been broken up mainly due to land use changes and drainage of breeding ponds. These processes have been ongoing but exacerbated during last 100 years. As seen in the selection analyses, adaptation to different local environments cannot be ruled out as a contributing cause of population differentiation.

The observed population differentiation in Sweden is likely caused by habitat fragmentation and local extinctions reducing connectivity. Within Sweden, there seem to be about five genetic clusters. The pattern is complicated by human-assisted translocations, many of which have been only partially successful. Hence, geographical distance is not necessarily the most accurate determinant of gene flow for the green toad. Utklippan appeared as genetically similar to Eskilstorp. The translocation, which most likely saved the Utklippan was supposedly done with animals from Eskilstorp and/or Limhamn (the records are uncertain). Our results suggest translocations from Eskilstorp were more successful. Falsterbo, Lundåkrabukten, and Norra hamnen clustered together in the PCA, showed similar admixture patterns, where Falsterbo and Lundåkrabukten appeared genetically similar to Perpparholmen. These subpopulations also show low pairwise genetic differentiation, indicating the existence of current or very recent gene flow. Their estimates of genetic diversity based on segregating sites were very similar; however, the Tajima’s D values differed in sign although were all close to zero.

### Differences in demographic histories

None of the subpopulations deviated significantly from neutral evolution expectations according to the Tajima’s D test statistic. High levels of observed heterozygosity and number of segregating sites being higher than the number of pairwise nucleotide differences would indicate an excess of rare alleles in a population, which would be expected following a founder event.^53^ The Lernacken, Norra hamnen, and Bornholm subpopulations in contrast, showed a positive Tajima’s D value indicating a scarcity of rare alleles. Generally, the genetic diversity measured by Watterson’s theta estimator was similar across all Swedish populations, yet Tajima’s D varied in magnitude and sign and did not deviate from zero. This implies that the overall low genetic diversity might be caused by a variety of demographic histories that have in part been influenced by human-mediated population support. Since among the Scandinavian populations analyzed here, there is a known decline and loss of connectivity, the expected signal is a positive sign of Tajima’s D. Translocations, however, can obscure such patterns leading to a false signal of population expansion.

### Inbreeding

ROH were used to estimate the level of past and present inbreeding, which impacts the risk of inbreeding depression and can therefore help guide conservation priorities and genetic rescue programs. A population with high homozygous proportions of the genome has an increased chance of suffering from negative fitness effects due to fixed, expressed, recessive, deleterious alleles, and lower evolutionary potential from the reduction of heterozygosity. In this system, we classified all ROH above 1 Mb as long runs that indicate more recent inbreeding. ROH between 100 kb and 1 Mb were assumed to result from historic inbreeding, where long runs have been broken up by recombination. It is important to note that our analysis likely provides a conservative estimate of ROH due to low sequencing depth. Sequencing errors can break up long ROH and easily remain undetected with only 5× genome coverage.

The Polish population had the lowest level of inbreeding measured by F_ROH_, much like what would be expected given the population’s higher genetic diversity. Limhamn, Brantevik, and Utklippan were the most “inbred” populations in Sweden with close to 40% of the genome made up of ROH. Limhamn had the highest number of ROH longer than 1 Mb indicating the most recent inbreeding, whereas Norra hamnen showed among the lowest levels of inbreeding. These results are most likely explained by genetic drift and historic inbreeding effects and have valuable implications for the ongoing species conservation action in Sweden suggesting Norra hamnen would be better suited as source for translocations than Limhamn. Furthermore, in line with the results on populations’ structure, it appears as if translocations have had limited impact on F_ROH_, again suggesting most of the released animals never survived to reproduction.

### Genetic load

Masked genetic load, regardless if highly deleterious or moderately so, is an order of magnitude higher than the realized load and that high impact mutations are less common than moderate impact mutations for both categories are not surprising. Purifying selection tends to remove homozygous high impact mutations faster than any mutation of lower impact. Furthermore, the impact of selection depends on the dominance relationships and any mutation that is completely masked (completely recessive) will be shielded from selection when heterozygous. Genetic load also depends on population size, where larger populations harbor more mutations, as exemplified by the Polish population, which has higher load than any of the Scandinavian populations.

A debate has ensued within the conservation genetics community, which donor populations to use when there are differently sized donor populations to choose from.^54^ Translocations from a large population will introduce more variation but inevitably comes at the cost of increasing the genetic load.^36^^,^^55^^,^^56^ Our results suggest that in order to avoid introducing load, Poland should be avoided. The other Scandinavian populations are quite similar and Limhamn and Norra hamnen might be the most suitable. However, more data on the specifics of the genetic load in any potential donor population are needed before firm conclusions can be drawn.

### Possible local adaptation

The Limhamn population has been used as the primary source for the breeding program and translocations as it is one of the largest natural populations and is free of the amphibian chytrid fungus *Batrachochytrium dendrobatitis* (Bd).^57^ The locality in Limhamn is a huge dug out and now abandoned lime stone quarry with a different microclimate and salinity levels compared to most other localities, likely resulting in divergent selection pressures on the Limhamn animals.^50^ The Limhamn population shows a putative signal of local adaptation based on outlier analyses. This may suggest the Limhamn population has responded to a different pH, salinity regime, and disease exposure compared to the other Scandinavian populations.

Our finding in the Gene Ontology enrichment analyses that genes involved in embryonal development show a signal of directional selection, suggests local adaptation in the Limhamn population. In particular, we got hits on genes involved in epiboly, cell movement in the gastrulation stage of embryonic development in amphibians and some other vertebrates, and morphogenesis. Larval development time is an important life history trait in amphibians, which has been found to be under divergent selection.^58^ We speculate that in the Limhamn alkaline environment with fewer food sources, higher mean temperatures and reduced competition,^50^^,^^57^ optimal larval development time and growth may be different from the other populations. Earlier studies have found high plasticity of the green toad to cope with variation in salinity and temperature, supporting the use of Limhamn as a source population.^50^ However, our outlier analysis indicate that the Limhamn population may have adapted to the special conditions in the abandoned lime stone quarry, although experimental evidence would be required to show this. In any case, an elevated level of inbreeding combined with a low level of genetic diversity, suggests considering alternative populations for further translocations.

### Conservation implications

Norra hamnen could provide a suitable alternative source population. It is a relatively large, admixed population with relatively high genetic diversity and the low proportion of inbreeding and moderate levels of genetic load. Nordens Ark moved 5 pairs from Norra hamnen to the captive breeding facility in May 2021. Offspring crosses from these individuals have been translocated as mixed (Limhamn × Norra Hamnen) to northern Öland in 2024 and 2025 as well as pure Norra hamnen to the island of Gotland in 2025. To strengthen the demographics and genetics, additional toads were collected from Norra hamnen in 2025. One concern when using more genetically diverse populations for translocations is the risk of outbreeding depression. The success of translocation projects is highly context dependent and can thus be difficult to predict. It is important to consider that when introducing individuals harboring high heterozygosity to a small, inbred population for genetic rescue, it is possible that the outbred immigrants introduce a masked mutational load that becomes exposed by inbreeding and can have detrimental effects on the population.^16^^,^^54^^,^^59^ In such a case, the attempt to avoid inbreeding depression results in outbreeding depression with negative fitness effects. To investigate these possible negative effects, we performed experimental crosses between individuals from Norra hamnen and Limhamn but are unfortunately lacking fitness data on these crosses. The crosses were perfectly intermediate between the two parental populations both in the PCA and admixture analysis and had the highest number of segregating sites among the Swedish populations. The hybrids showed similar levels as Norra hamnen for masked load and the success of the Norra hamnen population as one of the largest at present in Sweden, suggests that a high degree of admixture has not been detrimental for population growth in this case. Nevertheless, we are still lacking fitness data to properly assess the potential for successful translocation. The recent fragmentation of green toad populations in Sweden and nearby Denmark has led to genetic erosion within subpopulations meaning that many subpopulations contain unique variation. To save this variation all the remaining subpopulations should be a prime consideration of future conservation actions aimed at this species.

The Swedish green toad populations are small and fragmented with limited gene flow and low overall genetic diversity and they continue to decline despite extensive conservation efforts.^38^ To achieve the action plan vision of re-establishing a long-term viable metapopulation system across southern Sweden with at least 1,500 reproductive females, careful consideration of the genetic compatibilities and interactions between genetics and demography is advisable. From a conservation genomics perspective, our results suggest that the Limhamn population does not represent the most suitable source for translocations and we recommend considering Norra hamnen as a more favorable alternative.

### Limitations of the study

There is no single solution to the genetic rescue dilemma. It is difficult to decide between increasing genetic diversity and evolutionary potential on one hand and risking loss of local adaptation and possibly introducing masked genetic load on the other hand.^14^^,^^23^^,^^60^^,^^61^ A small recipient population that is inbred might be able to purge deleterious alleles; however, this would decrease adaptive potential, which seems especially problematic for a species with limited dispersal radius in times of rapidly changing climatic conditions. Ideally, when deciding on conservation management actions, there would be access to more information about mutation rates, types of mutations (loss of function, missense, and synonymous), mating system, and historic effective population sizes of the donor and recipient populations. However, for many threatened species it is difficult and cost-intensive to obtain such a comprehensive dataset. Performing whole-genome sequencing and estimating diversity parameters and levels of inbreeding based on genotype likelihoods is an important first step for the assessment of conservation actions.

## Resource availability

### Lead contact

Further information and requests for resources and reagents should be directed to and will be fulfilled by the lead contact Jacob Höglund, jacob.hoglund@ebc.uu.se.

### Materials availability

The study did not generate new unique reagents or restrictions to availability.

### Data and code availability

• The raw sequencing data are uploaded on European Nucleotide Archive (ENA) study accession no. PRJEB108485.
• All the scripts used for bioiniformatics and data analysis are available at PRMs github (https://github.com/PatrikRodinMorch/green_toad_low_coverage_conservation_genomics_project)

## Acknowledgments

This study was supported by a grant from the 10.13039/501100001862Swedish Research Council Formas, grant no. 2023-01150 (to J.H.) and the 10.13039/501100001725Royal Swedish Academy of Sciences (to P.R.-M.). The authors acknowledge support from the Danish Environmental Protection Agency and the county administration boards in Skåne and Blekinge counties for sampling permissions. Ethics permit for keeping and breeding animals at Nordens Ark were given by Gothenburg District Court. The National Genomics Infrastructure in Stockholm funded by 10.13039/501100009252Science for Life Laboratory, 10.13039/501100004063Knut and Alice Wallenberg Foundation, and the 10.13039/501100004359Swedish Research Council, and NAISS are acknowledged for assistance with massively parallel sequencing and access to the 10.13039/501100015701UPPMAX computational infrastructure.

## Author contributions

Conceptualization, P.R.-M and J.H.; methodology, A.F., M.S., M.W., P.R.-M., and J.H.; formal analysis, L.M.W., A.W.S., and P.R.-M.; investigation, L.M.W., A.W.S., M.S., A.F., M.W., R.D., and K.F.; resources, J.H.; data curation, P.R.-M., J.H., and M.W.; writing original draft, L.M.W. and J.H.; visualization and supervision, P.R.-M. and J.H.; project administration and funding acquisition, J.H.; writing – review and editing, all members.

## Declaration of interests

The authors declare no competing interests.

## Declaration of generative AI and AI-assisted technologies in the writing process

The authors did not use AI-assistance during the writing process except for shortening the title and reducing the abstract to 150 words.

## STAR★Methods

### Key resources table

**Table:** 

REAGENT or RESOURCE	SOURCE	IDENTIFIER
**Deposited data**		

Sequencing data	This study	European Nucleotide Archive (ENA) study accession: PRJEB108485

**Software and algorithms**		

FastQC v.0.11.9	Andrews^62^	https://www.bioinformatics.babraham.ac.uk/projects/fastqc/
Trimmomatic v 0.39	Bolger et al.^63^	https://github.com/usadellab/Trimmomatic
BWA v.0.7.17	Li & Durbin^64^	https://github.com/lh3/bwa
SAMtools v.1.2	Li et al.^65^	https://github.com/samtools/samtools
Picard v.3.1.1	Broad Institute	http://broadinstitute.github.io/picard
ANGSD v.0940	Korneliussen et al.^66^	https://www.popgen.dk/angsd/index.php/ANGSD
ngsLD v.1.2.0	Fox et al.^67^	https://github.com/fgvieira/ngsLD
prune_graph v.0.3.4	–	https://github.com/fgvieira/prune_graph
R v.4.5.1	R core team 2025	https://www.r-project.org/
ggplot2 v.3.5.2	Wickham^68^	https://ggplot2.tidyverse.org/
PCAngsd v1.36.4	Meisner & Albrechtsen^69^	https://github.com/Rosemeis/pcangsd
NGSadmix	Skotte et al.^70^	https://www.popgen.dk/software/index.php/NgsAdmix
CLUMPAK	Kopelman et al.^71^	https://clumpak.evolseq.net/
vcftools v.0.1.16	Danecek et al.^66^	https://vcftools.github.io/index.html
bcftools 1.19	Narasimhan et al.^72^	https://samtools.github.io/bcftools/bcftools.html
SnpEff v5.2	Cingolani et al.^73^	http://pcingola.github.io/SnpEff/
SnpSift v5.2	Cingolani et al.^73^	https://pcingola.github.io/SnpEff/snpsift/introduction/
Liftoff v.1.6.3	Shumate and Salzberg^74^	https://github.com/agshumate/Liftoff
q-value package v.2.40.0	Storey et al.^75^	https://www.bioconductor.org/packages/release/bioc/html/qvalue.html
CMplot v.4.5.1	Yin et al.^76^	https://github.com/YinLiLin/CMplot
bedtools v. 2.27.1	Quinlan and Hall^77^	https://bedtools.readthedocs.io/en/latest/
ShinyGO v.0.82	Ge et al.^78^	https://bioinformatics.sdstate.edu/go/
Analysis scripts	This study	https://github.com/PatrikRodinMorch/green_toad_low_coverage_conservation_genomics_project

### Method details

#### Information on the Scandinavian samples and sites used in the study

The translocation history of each subpopulation was extracted from a previous study^39^ and is summarized in Table S1.

Samples were collected from individual egg strings and raised to the first larval stage at ten sites in southern Sweden, two sites in Denmark (Figure 1) and one site in Poland between 2020 and 2024. Samples from a Polish site near Kuków was included as a reference of a population situated closer to the center of the mainland European green toad distribution. The Swedish samples were collected from all known sites with a breeding population. The Limhamn population serves as the source population for a captive breeding project at Nordens Ark Zoo which has been used for support releases during the last three decades (see below). For this paper we made experimental crosses between Limhamn and Norra hamnen to test for possible outbreeding depression (four families). The sequencing data is comprised of one to eight individuals per population for a total of 78 samples (Table 1). We merged two samples from Hylkan with nearby Brantevik as preliminary analyses made it apparent that these individuals originated from Brantevik.

Genomic DNA was extracted from larvae using an in-house salt extraction protocol (modified after^79^) and sent for library preparation and sequencing to NGI (National Genomics Infrastructure) at SciLifeLab, Solna. The Illumina DNA PCR-free kit was used to prepare libraries from 65 to 40 genomic DNAs, with 200 ng gDNA as input. The libraries were pooled and sequenced on two Illumina NovaSeq 6000 S4-300 lanes and on one Illumina NovaSeq XPlus 25B-300 lane, with a PE150 read setup and an average of 70M read-pairs (21 Gb) or 90M read-pairs (27 Gb) of data per sample. The whole-genome re-sequencing data had an individual average depth of coverage of approximately 5× – 11×.

#### Identifying genetic variants

The quality of the raw reads was assessed using FastQC v.0.11.9^62^ and Illumina sequencing adapters were removed using Trimmomatic v 0.39.^63^ The sequence reads were aligned to a Swedish green toad reference genome^80^ using BWA mem v.0.7.17^64^ with default settings. The reads were sorted by genomic position using SAMtools v.1.2^65^ and duplicate reads were identified with the Picard MarkDuplicates v.3.1.1 (http://broadinstitute.github.io/picard). ANGSD v.0940^66^ was used to estimate variants based on genotype likelihoods (GL) and thus accounted for the uncertainty of low coverage genomic data. The genotype likelihoods were obtained using the Genome Analysis Toolkit (GATK) likelihood model (-GL 2).^81^ The program estimated the major and minor alleles for each site and the minor allele frequency based on the calculated genotype likelihoods from the sample BAM files with a minimum mapping quality (mapQ) set to 30 and base quality (minQ), and only variants with a minor allele frequency of 5% and a *p*-value of 1e-6 and below were deemed significant. The genotype likelihood output was stored in beagle likelihood format (-doGlf 2). The data was subsetted to investigate different hierarchies of population structure and putative selection. In all analyses not related to those topics the full dataset was used. For the dataset containing all populations we obtained 24,447,687, for Scandinavia 22,979,083 and for the selection scan (methods detailed below) 22,475,893 variant sites.

### Quantification and statistical analyses

#### Calculations of population structure

As the methods used here to detect population structure assumes unlinked variants to a certain extent, we pruned all three datasets described above based on linkage disequilibrium (LD). LD pruning was conducted using ngsLD v.1.2.0^67^ to estimate pairwise r^2^ with a maximum distance between two variants set to 100 Kb. Due to the computational cost and the size of the final LD file we only retained 5% of all pairwise comparisons. The three datasets were then pruned using prune_graph v.0.3.4 (https://github.com/fgvieira/prune_graph) with a maximum r^2^ allowed between two variants set to 0.5. This resulted in a total of 13,355,921 unlinked SNPs for the dataset containing all populations, 12,774,506 for the Swedish populations and 12,431,821 for the dataset used in the selection scan.

Unless otherwise stated, all plots were created using ggplot2 v.3.5.2^68^ in R v.4.5.1. The analyses of population structure and admixture proportions were performed in PCAngsd v1.36.4.^69^ First, to assess population structure a PCA was generated from eigenvectors of the estimated covariance matrix. Secondly, Admixture proportions^70^^,^^71^ across 10 independent runs were visualized using CLUMPAK^70^ and the optimal value of K was plotted in ggplot2.admixture proportions were estimated using NGSadmix^70^ for K number of clusters ranging from 2 to 12.

#### Calculations of genetic diversity and differentiation

We used the folded site frequency spectrum (SFS) for estimating observed heterozygosity (H_O_), Watterson’s theta (θ_W_), nucleotide diversity (θ_π_) and Tajima’s D.^82^ The -dosaf flag in ANGSD was used to find a global estimate of SFS for each population. It applies maximum likelihood statistics (maximizing the probability of the allele frequency, given the data) to estimate the SFS based on the genotype likelihood at each site, assuming Hardy Weinberg Equilibrium.^82^

We computed the genome-wide pairwise F_ST_ values between all populations based on the folded site allele frequency (saf) files for each population. ANGSD calculated the 2days site frequency spectrum for each of the 28 pairs using the realSFS command. The joint sfs files and individual population saf files were then used to generate pairwise F_ST_ values. The -whichFst 1 command is recommended for small sample sizes as it is bias-corrected and more stable to differences in sample size.^83^

#### Calculations of inbreeding through F_ROH_

Even though it is not generally recommended to use hard called single nucleotide polymorphisms (SNPs) genotypes from low coverage genomic data due to high statistical uncertainty,^69^ we used called genotypes for runs of homozygosity (ROH) and genetic load estimates, with the motivation that since we are interested in the relative differences between the populations no single population should experience any systematic bias if all individuals have the same low coverage. Therefore, we sub-sampled the bam files to a coverage of 5× using samtools v.1.2^65^ before calling genotypes, which was done using the following parameters in ANGSD -GL 2 -dopost 1 -domajorminor 1 -domaf 1 -dobcf 1 --ignore-RG 0 -dogeno 1 -docounts 1 -snp_pval 1e-6 -minMapQ 30 -minQ 20. The subsequent vcf was further filtered in vcftools v.0.1.16^66^ by setting a minimum and maximum depth of 2 and 15 respectively, allowing only biallelic sites and at most 10% missing data and a minor allele count of 2. This allowed 10,607,526 SNPs in the final dataset. To identify runs of homozygosity (ROH) we used the roh command with --GTs-only 30 in bcftools 1.19,^72^ that uses a hidden Markov model to identify homozygous stretches along the genome.^84^ The genomic inbreeding coefficient F_ROH_^20^ was calculated dividing the sum of total ROH length by the length of the autosomal genome (the size of the assembled genome) and averaged across each population. To allow differentiation between signatures of recent inbreeding and historical demography, each ROH was assigned to one of three size classes (100-500 kb, 500 kb-1Mb, >1 Mb) and the size distribution per individual and population was visualized.

#### Estimation of genetic load

The raw reads were re-aligned to a green toad reference assembly^85^ of an individual collected in Greece, following the same workflow as above. We assume that the reference variants are ancestral to our Northern European individuals in order to polarize our predicted deleterious variants.

To estimate genetic load we ran SnpEff v5.2a^73^ on a custom database built from the reference genome from the Greek individual to annotate each variant in the VCF. However, as the reference entry lacks an annotation file we performed annotation lift-over from the Swedish reference genome annotation using Liftoff v.1.6.3.^74^ SnpEff estimates variants to be of low (such as synonymous mutation), moderate (such as missense mutation) or high impact (such as premature stop codon or frameshift mutations) based on their predicted effect of the protein relative to the reference sequence used to build the snpEff database. Variants of these effects were then extracted from the VCF using SnpSift v5.2.^73^ The number of heterozygous and homozygous variants of each impact class was counted for each individual using a custom script. We define masked genetic load as the number of heterozygous “derived” moderate and high impact variants per population and realized load as the number of homozygous “derived” variants. The final dataset contained 14,057,104 SNPs.

#### Evidence of divergent selection and local adaptation

To identify signatures of putative divergent selection in the Limhamn source population compared to the other Scandinavian populations, we utilized the selection parameter in PCAngsd v1.11,^69^ that extends the model of FastPCA^74^ which attempts to identify selection at variants whose differentiation is greater than a null distribution of genetic drift. The selection statistics (chi2 distributed) were converted to *p*-values using the R function pchisq with 1° of freedom. We removed individuals from Bornholm as they have diverged substantially due to drift, and we removed the Limhamn-Norra hamnen hybrids. We only considered variants associated with PC3, as that axis is the main separator of the *ex situ* source population Limhamn with all other Swedish populations (Figure S2B). Additionally, no variants significantly associated with structure along PC1 (Figure S2B) were found indicating that there is no signal of putative local adaptation among the populations separated along the major axis of variation, however, 5618 variants were associated with PC2. The *p*-values values were converted to q-values to correct for multiple testing using the q-value package v.2.40.0.^86^ Q-values where visualized in a manhattan plot using the CMplot v.4.5.1.^75^ The positions of significant variants (q-value <0.01) were extracted and intersected with genes in the annotation file within 20 Kb in each direction, using bedtools window v. 2.27.1.^76^ Potential enrichment of gene ontology terms was assessed using ShinyGO v.0.82^77^ querying against *Xenopus tropicalis* ontology terms and a false discovery rate of 0.05.
