# Supplementary material for: Genomic insights into fragmentation and translocation in European green toads
Source: iScience. 2026 Mar 17;29(4):115395. doi: 10.1016/j.isci.2026.115395 (PMC13084336; doi:10.1016/j.isci.2026.115395)
Supplement: Document S1. Figures S1–S5 and Tables S1 and S2 [file mmc1.pdf]

iScience, Volume *29*

Supplemental information

Genomic insights into fragmentation and translocation in European green toads

Leonie Muriel Walderich, Alvin Wiwiet Susanto, Mikael Svensson, Anna Fohrman, Mats Wirén, Rachael O'Dwyer, Kristofer Försäter, Patrik Rödin-Mörch, and Jacob Höglund

Supplemental information

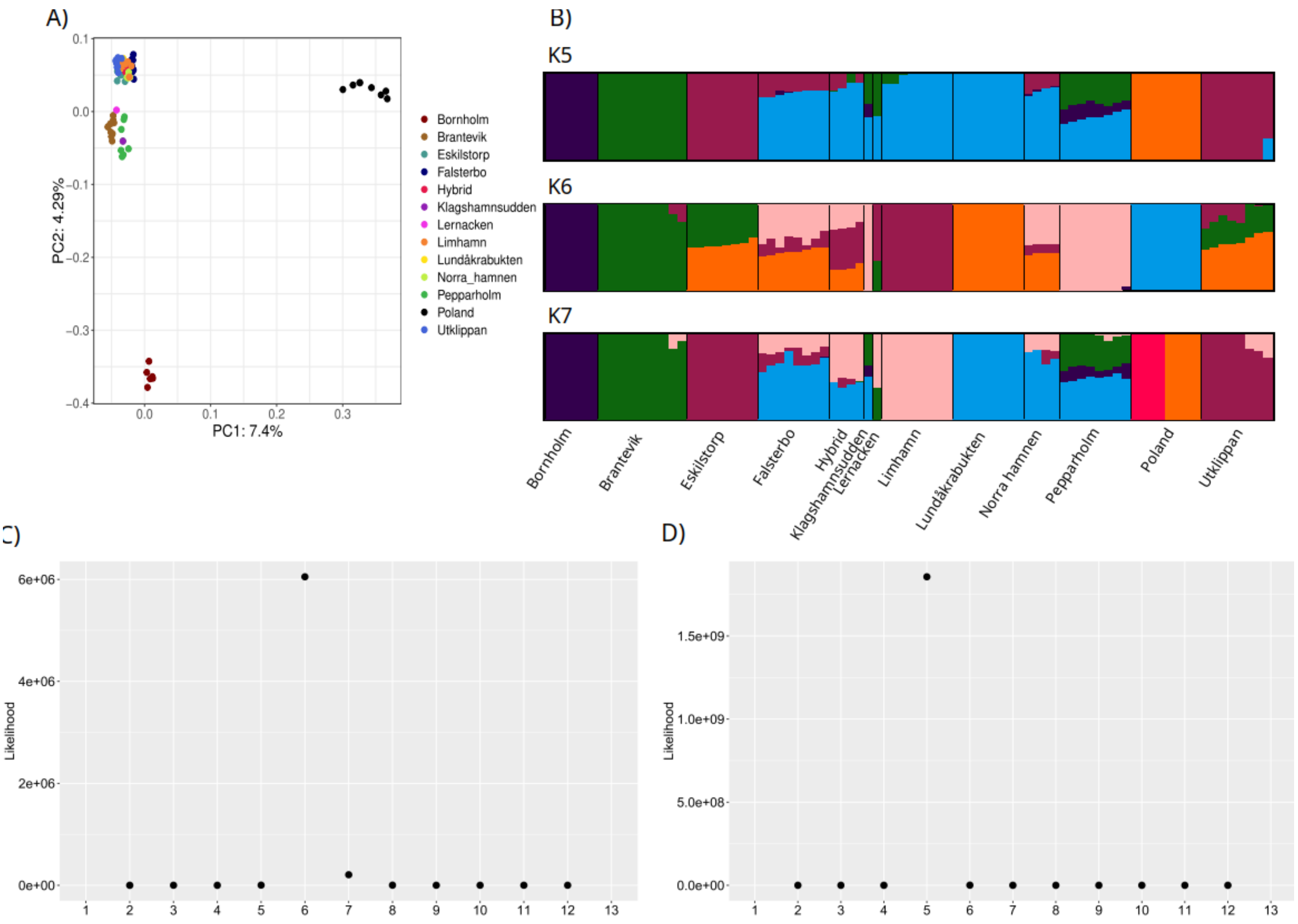

Figure S1. PCA plots of A) all samples and B) Admixture plots showing all samples including the Polish population, showing ancestry coefficients of each individual at K = 4-6, C) delta K values for the NGSadmix of all samples indicating K=6 as the optimal number, D) delta K values for only the Scandinavian samples indicating K=5 as the optimal number.

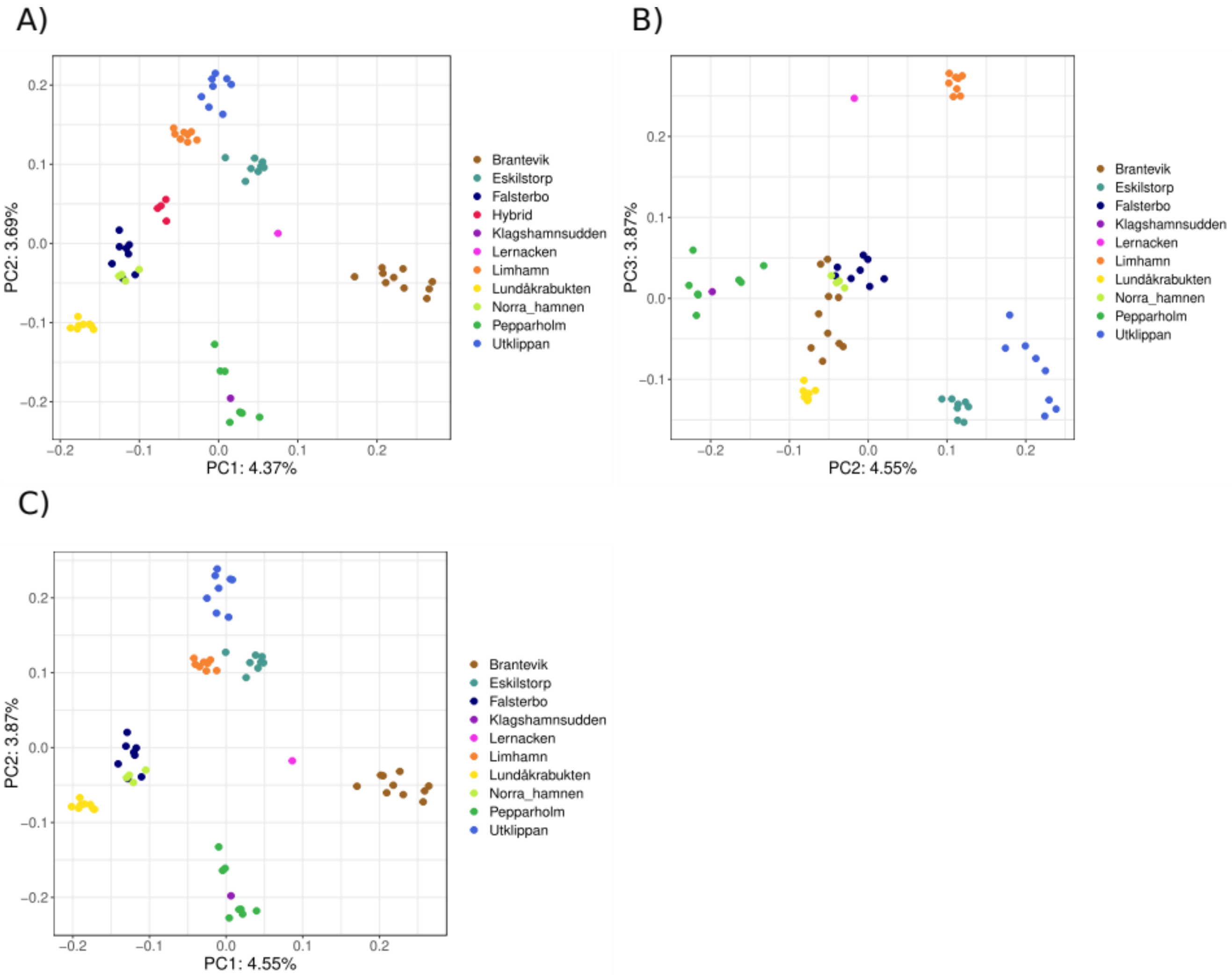

Figure S2. PCA plots of A) all Scandinavian samples excluding Bornholm including hybrids and B) the Scandinavian samples used for the selection scan. C) all Scandinavian samples excluding Bornholm and hybrids.

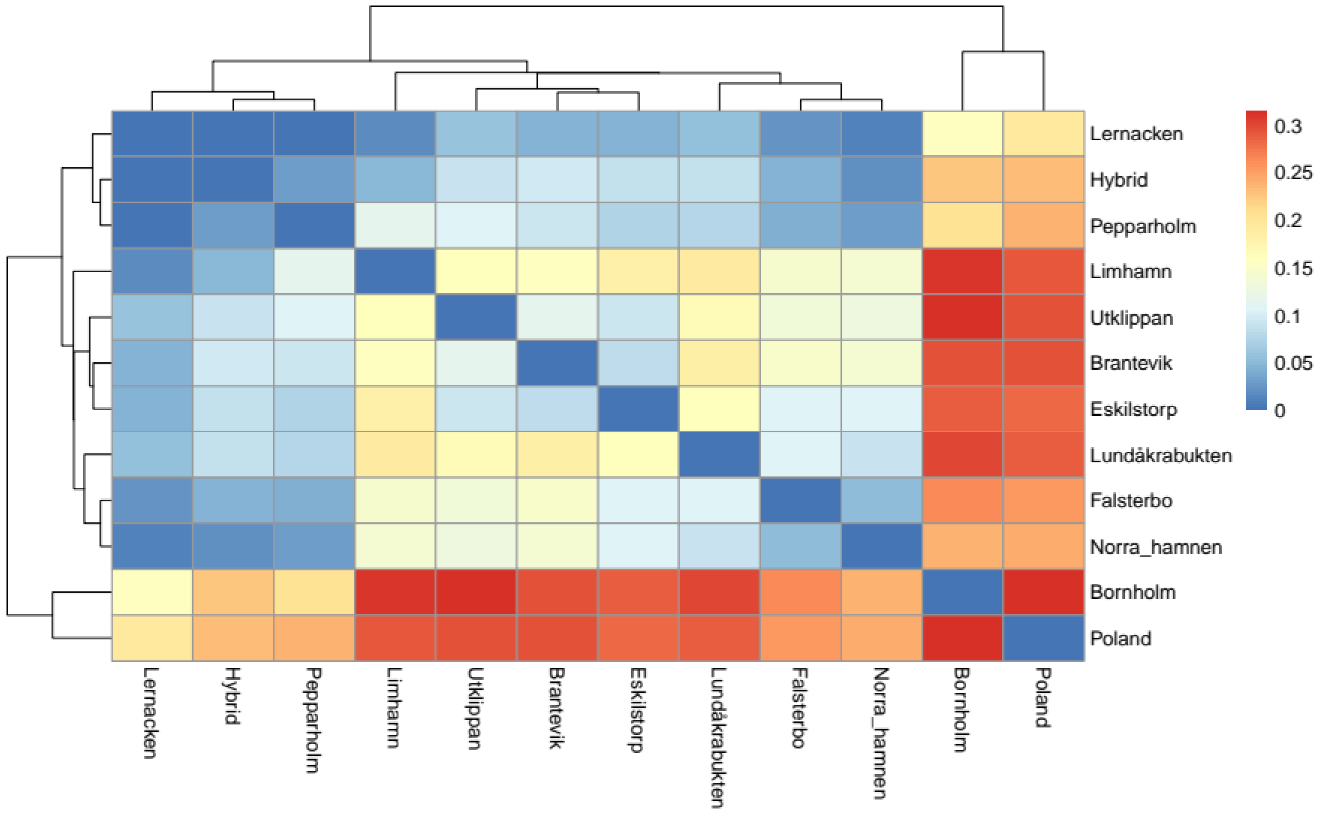

Figure S3. Heat map of the genome-wide pairwise F_{ST} values for each population comparison. The darker red fields represent higher F_{ST} values and thus a higher degree of genetic differentiation.

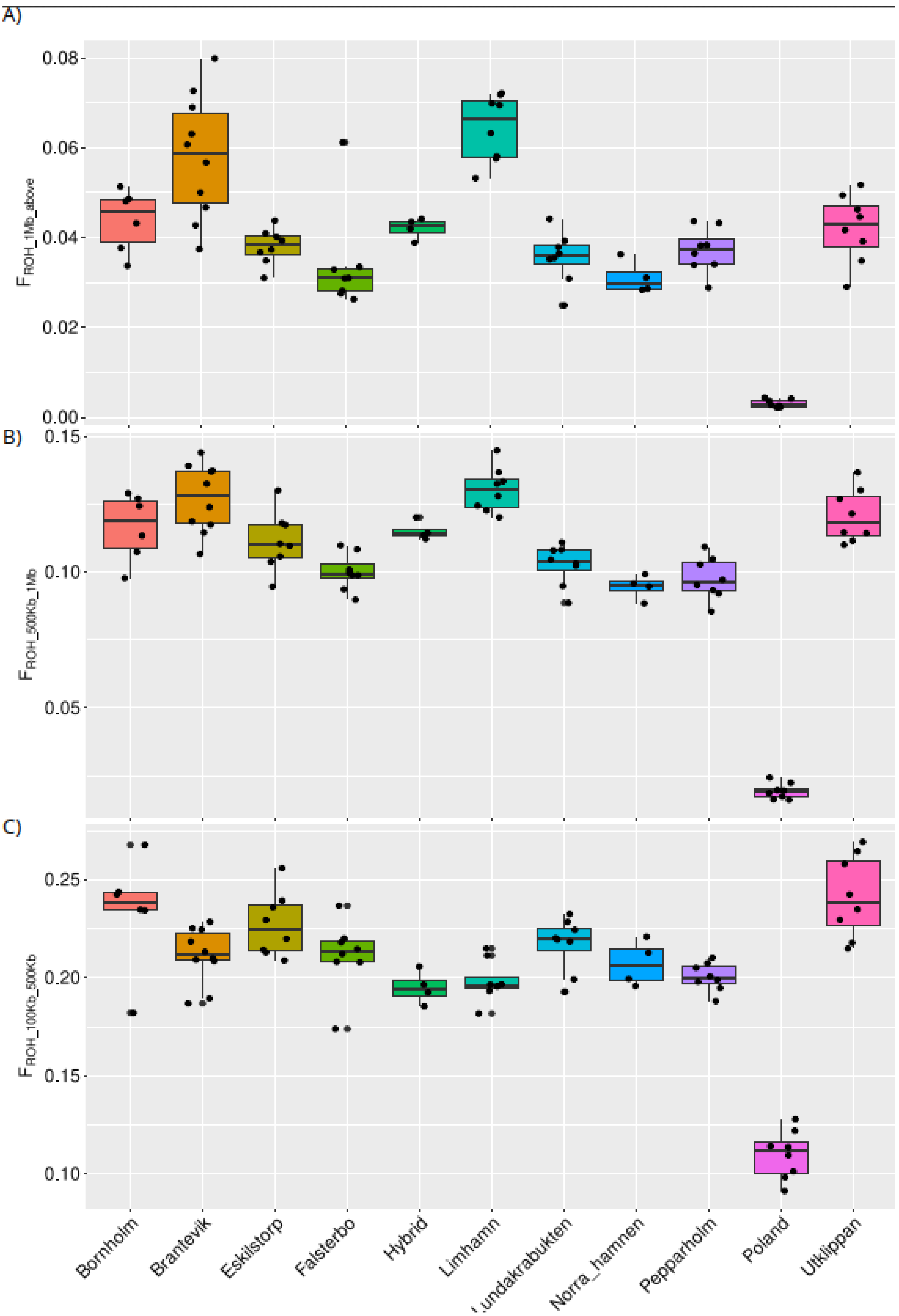

Figure S4. Median F_{ROH} (line through the box indicate the median, boxes describe 25-75 percentiles, outlier values are given as points) of all individuals in all populations in individual size classes a) >1Mb, b) 500 - >1 kb, c) 100-500 kb.

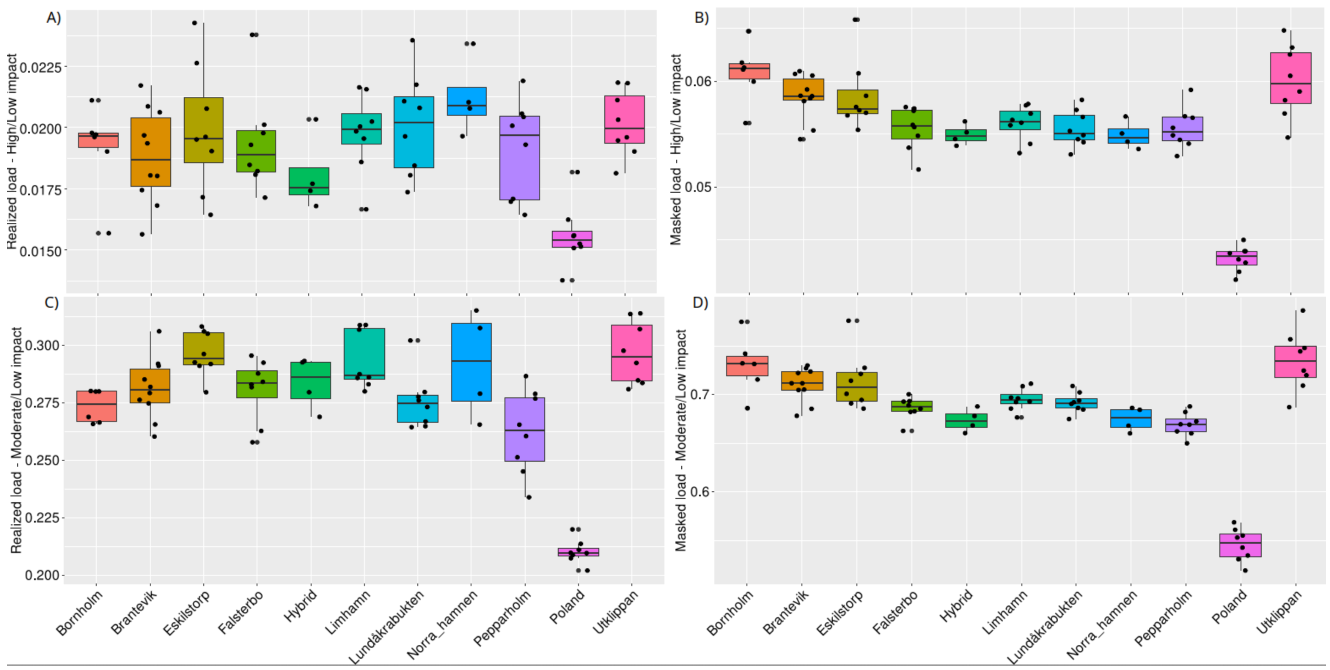

Figure S5. Boxplots showing the ratio of deleterious mutations to low impact synonymous mutations in each population (line through the box indicate the median counts, boxes describe 25-75 percentiles, outlier values are given as points). A) Counts of realized (HIGH impact variants, B) masked HIGH impact variants, C) Realized MODERATE impact variants, D) Masked MODERATE impact variants.

Table S1. Known translocations among contemporary populations 1994-2022 (LI-Limhamn. NA- Nordens Ark Zoo, ES-Eskilstorp, LN- Lernacken, NH- Norra hamnen, HB- Högaborg)

Locality	Source	Direct/Breeding	Year	Eggs	Larvae	Tadpoles	Juveniles 1-3w	Older > 4w
Brantevik	LI (NA)	Breeding	2006				650	
Brantevik	LI(ES?) (NA)	Breeding	2007				900	
Brantevik	LI+ES	Direct	2009		1 800	400		
Brantevik	LI+LN	Direct	2011	25 000	20 000			
Brantevik	LI	Direct	2012	35 000	3 800			
Brantevik	LI	Direct	2013		7 000			
Brantevik	LI	Direct	2014	14 000	4 000			
Eskilstorp	ES	Breeding	1997		142		242	
Eskilstorp	LI	Breeding	1999		1 000		10	
Eskilstorp	LI	Breeding	2000		6 200	605		
Eskilstorp	LI 85% ES 15%	Breeding	2001		9 400	451		
Eskilstorp	LI 85% ES 15%	Breeding	2002		28 800	448		
Falsterbo	LI	Breeding	2000		6 100			
Falsterbo	LI 85% ES 15%	Breeding	2002		19 200	252		
Klagshamnsudden	LI 85% ES 15%	Breeding	2001		8 150	415		
Klagshamnsudden	LI 85% ES 15%	Breeding	2002		19 200	350		
Klagshamnsudden	LI	Direct	2003					110
Klagshamnsudden	LN	Direct	2009		3 000			
Klagshamnsudden	LI	Direct	2010	12 000	2 800			
Klagshamnsudden	LI+LN	Direct	2011	5 000	30 000			
Klagshamnsudden	LN	Direct	2012		2 000			
Klagshamnsudden	LI	Direct	2015		2 500			
Klagshamnsudden	LI+NH	Direct	2016	600				80
Klagshamnsudden	NH	Direct	2017					307
Klagshamnsudden	NH	Direct	2019					41
Klagshamnsudden	LI (NA)	Breeding	2020			4 700	300	
Klagshamnsudden	NH	Direct	2022					55
Lernacken	LI	Direct	2004					183
Lernacken	LI	Direct	2005				1 290	
Lernacken	LI	Direct	2006				377	
Lernacken	LI 30%, ES 60%, HB 10%	Direct	2007	7 000			787	
Lernacken	ES	Direct	2008		1 500	255		
Lernacken	ES	Direct	2009		2 000			
Lernacken	LI	Direct	2013		1 000			
Lernacken	LI(NA)	Direct	2020			4 700	300	
Limhamn	LI	Breeding	1999		34	40	15	
Limhamn	LI 85% ES 15%	Breeding	2000		2 500	202		
Limhamn	LI 85% ES 15%	Breeding	2001		3 000	712		
Utklippan	LI+(ES?)	Breeding+Direct	1994				100	10

Table S2. ShinyGO significantly enriched gene ontology terms for biological processes, molecular function and cellular components.

Enrichment FDR	***nGenes***	***Pathway genes***	***Fold Enrichment***	
**Biological processes**				
6.4E-03	2	4	132.1	Wound healing spreading of cells
6.4E-03	2	4	132.1	Epiboly involved in wound healing
6.4E-03	2	4	132.1	Skeletal muscle satellite cell migration
1.3E-02	2	7	75.5	Epiboly
1.3E-02	2	9	58.7	Pos. reg. of cell projection organization
1.3E-02	2	9	58.7	Reg. of cell projection assembly
1.3E-02	2	9	58.7	Reg. of plasma membrane bounded cell projection assembly
1.8E-02	2	12	44	Morphogenesis of an epithelial sheet
6.4E-03	3	21	37.7	Reg. of cell projection organization
6.4E-03	3	21	37.7	Reg. of plasma membrane bounded cell projection organization
7.0E-03	3	25	31.7	Pos. reg. of cellular component organization
1.3E-02	4	86	12.3	Secretion by cell
4.1E-02	3	68	11.7	Exocytosis
1.3E-02	4	95	11.1	Export from cell
1.4E-02	4	103	10.3	Secretion
1.1E-02	5	145	9.1	Reg. of cellular component organization
1.4E-02	8	511	4.1	Cellular component assembly
1.3E-02	9	615	3.9	Cellular component biogenesis
1.3E-02	14	1371	2.7	Cellular component organization
1.6E-02	14	1474	2.5	Cellular component organization or biogenesis
**Molecular function**				
None				
**Cellular component**				
3.0E-02	3	34	22.4	Vesicle tethering complex
